# Supplementary material for: Degradation of High Energy Materials Using Biological Reduction: A Rational Way to Reach Bioremediation
Source: Int J Mol Sci. 2019 Nov 7;20(22):5556. doi: 10.3390/ijms20225556 (PMC6888211; doi:10.3390/ijms20225556)
Supplement: Supplementary file 1 [file ijms-20-05556-s001.pdf]

Supplementary Materials

Scripts and param files required for the design phase

Param files of the cofactor FMNH2

```
NAME FMH2
IO_STRING FMH2 Z
TYPE LIGAND
AA UNK
ATOM  C14 CH1   X   0.00
ATOM  C13 CH2   X   0.00
ATOM  N4  Npro  X   1.00
ATOM  C11 aroC   X   0.00
ATOM  C4  aroC  X   0.00
ATOM  N3  Ntrp  X   1.00
ATOM  C3  aroC  X   0.00
ATOM  C2  CNH2  X   0.00
ATOM  N2  Ntrp  X   0.00
ATOM  C1  aroC  X   0.00
ATOM  N1  Ntrp  X   0.00
ATOM  C12 aroC  X   0.00
ATOM  H1  Hpol  X   0.00
ATOM  O1  ONH2  X   0.00
ATOM  H3  Hpol  X   0.00
ATOM  O2  ONH2  X   0.00
ATOM  H2  Hpol  X   0.00
ATOM  C5  aroC  X   0.00
ATOM  C6  aroC  X   0.00
ATOM  C7  CH3   X   0.00
ATOM  H5  Hapo  X   0.00
ATOM  H6  Hapo  X   0.00
ATOM  H7  Hapo  X   0.00
ATOM  C8  aroC  X   0.00
ATOM  C9  CH3   X   0.00
ATOM  H8  Hapo  X   0.00
ATOM  H9  Hapo  X   0.00
ATOM  H10 Hapo  X   0.00
ATOM  C10 aroC  X   0.00
ATOM  H11 Haro  X   0.00
ATOM  H4  Haro  X   0.00
ATOM  H12 Hapo  X   0.00
ATOM  H13 Hapo  X   0.00
ATOM  O3  OH    X   0.00
ATOM  H15 Hpol  X   0.00
ATOM  C15 CH1   X   0.00
ATOM  O4  OH    X   0.00
ATOM  H17 Hpol  X   0.00
ATOM  C16 CH1   X   0.00
ATOM  O5  OH    X   0.00
ATOM  H19 Hpol  X   0.00
ATOM  C17 CH2   X   0.00
```

```
ATOM  O6  OH    X   0.00
ATOM  P1  Phos  X   0.00
ATOM  O7  OOC   X   0.00
ATOM  O8  OH    X   0.00
ATOM  H22 Hpol  X   0.00
ATOM  O9  OH    X   0.00
ATOM  H23 Hpol  X   0.00
ATOM  H20 Hapo  X   0.00
ATOM  H21 Hapo  X   0.00
ATOM  H18 Hapo  X   0.00
ATOM  H16 Hapo  X   0.00
ATOM  H14 Hapo  X   0.00
BOND_TYPE  N1   C1  4
BOND_TYPE  N1   C12 1
BOND_TYPE  N1   H1  1
BOND_TYPE  C1   O1  2
BOND_TYPE  C1   N2  4
BOND_TYPE  N2   C2  4
BOND_TYPE  N2   H3  1
BOND_TYPE  C2   O2  2
BOND_TYPE  C2   C3  1
BOND_TYPE  C3   N3  4
BOND_TYPE  C3   C12 4
BOND_TYPE  N3   C4  4
BOND_TYPE  N3   H2  1
BOND_TYPE  C4   C5  4
BOND_TYPE  C4   C11 4
BOND_TYPE  C5   C6  4
BOND_TYPE  C5   H4  1
BOND_TYPE  C6   C7  1
BOND_TYPE  C6   C8  4
BOND_TYPE  C7   H5  1
BOND_TYPE  C7   H6  1
BOND_TYPE  C7   H7  1
BOND_TYPE  C8   C9  1
BOND_TYPE  C8   C10 4
BOND_TYPE  C9   H8  1
BOND_TYPE  C9   H9  1
BOND_TYPE  C9   H10 1
BOND_TYPE  C10  C11 4
BOND_TYPE  C10  H11 1
BOND_TYPE  C11  N4  4
BOND_TYPE  N4   C12 4
BOND_TYPE  N4   C13 1
BOND_TYPE  C13  C14 1
BOND_TYPE  C13  H12 1
BOND_TYPE  C13  H13 1
BOND_TYPE  C14  O3  1
BOND_TYPE  C14  C15 1
BOND_TYPE  C14  H14 1
BOND_TYPE  O3   H15 1
```

```
BOND_TYPE  C15  O4  1
BOND_TYPE  C15  C16 1
BOND_TYPE  C15  H16 1
BOND_TYPE  O4   H17 1
BOND_TYPE  C16  O5  1
BOND_TYPE  C16  C17 1
BOND_TYPE  C16  H18 1
BOND_TYPE  O5   H19 1
BOND_TYPE  C17  O6  1
BOND_TYPE  C17  H20 1
BOND_TYPE  C17  H21 1
BOND_TYPE  O6   P1  1
BOND_TYPE  P1   O7  2
BOND_TYPE  P1   O8  1
BOND_TYPE  P1   O9  1
BOND_TYPE  O8   H22 1
BOND_TYPE  O9   H23 1
CHI 1  C13  C14  O3   H15
PROTON_CHI 1 SAMPLES 3 60 -60 180 EXTRA 0
CHI 2  C14  C15  O4   H17
PROTON_CHI 2 SAMPLES 3 60 -60 180 EXTRA 0
CHI 3  C15  C16  O5   H19
PROTON_CHI 3 SAMPLES 3 60 -60 180 EXTRA 0
CHI 4  O6   P1   O8   H22
PROTON_CHI 4 SAMPLES 2 0 180 EXTRA 0
CHI 5  O6   P1   O9   H23
PROTON_CHI 5 SAMPLES 2 0 180 EXTRA 0
CHI 6  C14  C13  N4   C11
CHI 7  O3   C14  C13  N4
CHI 8  C13  C14  C15  O4
CHI 9  C14  C15  C16  O5
CHI 10  C15  C16  C17  O6
CHI 11  C16  C17  O6   P1
CHI 12  C17  O6   P1   O7
NBR_ATOM  C14
NBR_RADIUS 10.218452
ICOOR_INTERNAL    C14    0.000000    0.000000    0.000000   C14   C13   N4
ICOOR_INTERNAL    C13    0.000000  180.000000    1.555212   C14   C13   N4
ICOOR_INTERNAL    N4     0.000000   69.018287    1.483513   C13   C14   N4
ICOOR_INTERNAL    C11  -69.571570   59.741202    1.404395   N4    C13   C14
ICOOR_INTERNAL    C4  -177.329896   60.312591    1.390264   C11   N4    C13
ICOOR_INTERNAL    N3     0.021972   59.733563    1.348364   C4    C11   N4
ICOOR_INTERNAL    C3     8.101265   59.159442    1.361276   N3    C4    C11
ICOOR_INTERNAL    C2   173.222327   58.480227    1.396286   C3    N3    C4
ICOOR_INTERNAL    N2   179.849720   60.429160    1.353535   C2    C3    N3
ICOOR_INTERNAL    C1    -1.299891   59.495697    1.361523   N2    C2    C3
ICOOR_INTERNAL    N1     0.846183   59.229154    1.359719   C1    N2    C2
ICOOR_INTERNAL    C12    1.041603   59.157064    1.353604   N1    C1    N2
ICOOR_INTERNAL    H1  -179.973987   60.379495    1.008974   N1    C1    C12
ICOOR_INTERNAL    O1   178.452117   59.761543    1.247473   C1    N2    N1
ICOOR_INTERNAL    H3  -179.991253   60.772242    0.970007   N2    C2    C1
```

```
ICOOR_INTERNAL   O2  -178.821769   58.539052   1.256523   C2   C3   N2
ICOOR_INTERNAL   H2  -179.977962   60.435525   1.009598   N3   C4   C3
ICOOR_INTERNAL   C5   171.112181   61.337460   1.399196   C4   C11  N3
ICOOR_INTERNAL   C6     3.148550   58.079171   1.388951   C5   C4   C11
ICOOR_INTERNAL   C7   177.840913   59.485306   1.505838   C6   C5   C4
ICOOR_INTERNAL   H5    89.910379   70.461961   1.090285   C7   C6   C5
ICOOR_INTERNAL   H6   120.086060   70.498863   1.089839   C7   C6   H5
ICOOR_INTERNAL   H7   119.938582   70.526733   1.089995   C7   C6   H6
ICOOR_INTERNAL   C8   179.408417   60.709682   1.387086   C6   C5   C7
ICOOR_INTERNAL   C9  -178.498822   57.768278   1.507009   C8   C6   C5
ICOOR_INTERNAL   H8    89.979488   70.472616   1.090406   C9   C8   C6
ICOOR_INTERNAL   H9   120.075554   70.529056   1.091088   C9   C8   H8
ICOOR_INTERNAL   H10  119.934038   70.516082   1.090943   C9   C8   H9
ICOOR_INTERNAL   C10  178.199634   60.954718   1.378101   C8   C6   C9
ICOOR_INTERNAL   H11 -177.244146   59.904714   1.080178   C10  C8   C6
ICOOR_INTERNAL   H4   179.841485   59.980489   1.080686   C5   C4   C6
ICOOR_INTERNAL   H12 -119.987626   70.526257   1.090834   C13  C14  N4
ICOOR_INTERNAL   H13 -119.941887   70.538864   1.089687   C13  C14  H12
ICOOR_INTERNAL   O3   -78.827378   67.163186   1.431311   C14  C13  N4
ICOOR_INTERNAL   H15   -0.053087   66.003659   0.966091   O3   C14  C13
ICOOR_INTERNAL   C15 -123.479490   70.689407   1.540697   C14  C13  O3
ICOOR_INTERNAL   O4   -64.167402   73.183673   1.428152   C15  C14  C13
ICOOR_INTERNAL   H17    0.044109   65.995506   0.966894   O4   C15  C14
ICOOR_INTERNAL   C16 -121.268473   63.864088   1.523201   C15  C14  O4
ICOOR_INTERNAL   O5   -65.113033   69.820883   1.420679   C16  C15  C14
ICOOR_INTERNAL   H19   -0.053710   66.038203   0.967349   O5   C16  C15
ICOOR_INTERNAL   C17 -120.187930   69.030778   1.536748   C16  C15  O5
ICOOR_INTERNAL   O6    57.117293   70.489505   1.434048   C17  C16  C15
ICOOR_INTERNAL   P1  -117.575810   56.527066   1.607340   O6   C17  C16
ICOOR_INTERNAL   O7    55.452758   70.540109   1.480367   P1   O6   C17
ICOOR_INTERNAL   O8   119.924363   70.581162   1.609793   P1   O6   O7
ICOOR_INTERNAL   H22    0.057011   65.940809   0.966626   O8   P1   O6
ICOOR_INTERNAL   O9   120.015173   72.708547   1.514599   P1   O6   O8
ICOOR_INTERNAL   H23 -179.971219   65.980507   0.966319   O9   P1   O6
ICOOR_INTERNAL   H20  120.006632   70.537714   1.090591   C17  C16  O6
ICOOR_INTERNAL   H21  120.054734   70.530060   1.089849   C17  C16  H20
ICOOR_INTERNAL   H18 -119.848924   70.545264   1.090123   C16  C15  C17
ICOOR_INTERNAL   H16 -118.711138   70.613268   1.089718   C15  C14  C16
ICOOR_INTERNAL   H14 -116.556872   70.479723   1.090584   C14  C13  C15
```

Param files of the ligand HMX

```
IO_STRING HMX  Z
TYPE LIGAND
AA UNK
ATOM  C2  CH2   X  0.13
ATOM  N1  Nhis  X  -0.22
ATOM  N5  Npro  X  -0.06
ATOM  O1  OOC   X  -0.45
ATOM  O5  ONH2  X  -0.24
ATOM  C1  CH2   X  0.13
ATOM  N2  Nhis  X  -0.22
```

```
ATOM  N6  Npro  X   -0.06
ATOM  O2  OOC   X   -0.45
ATOM  O6  ONH2  X   -0.24
ATOM  C3  CH2   X   0.13
ATOM  N4  Nhis  X   -0.22
ATOM  N8  Npro  X   -0.06
ATOM  O4  OOC   X   -0.45
ATOM  O8  ONH2  X   -0.24
ATOM  C4  CH2   X   0.13
ATOM  N3  Nhis  X   -0.22
ATOM  N7  Npro  X   -0.06
ATOM  O3  OOC   X   -0.45
ATOM  O7  ONH2  X   -0.24
ATOM  H7  Hapo  X   0.41
ATOM  H8  Hapo  X   0.41
ATOM  H5  Hapo  X   0.41
ATOM  H6  Hapo  X   0.41
ATOM  H1  Hapo  X   0.41
ATOM  H2  Hapo  X   0.41
ATOM  H3  Hapo  X   0.41
ATOM  H4  Hapo  X   0.41
BOND_TYPE  O1   N5  1
BOND_TYPE  O2   N6  1
BOND_TYPE  O3   N7  1
BOND_TYPE  O4   N8  1
BOND_TYPE  O5   N5  2
BOND_TYPE  O6   N6  2
BOND_TYPE  O7   N7  2
BOND_TYPE  O8   N8  2
BOND_TYPE  N1   N5  1
BOND_TYPE  N1   C1  1
BOND_TYPE  N1   C2  1
BOND_TYPE  N2   N6  1
BOND_TYPE  N2   C1  1
BOND_TYPE  N2   C3  1
BOND_TYPE  N3   N7  1
BOND_TYPE  N3   C2  1
BOND_TYPE  N3   C4  1
BOND_TYPE  N4   N8  1
BOND_TYPE  N4   C3  1
BOND_TYPE  N4   C4  1
BOND_TYPE  C1   H1  1
BOND_TYPE  C1   H2  1
BOND_TYPE  C2   H3  1
BOND_TYPE  C2   H4  1
BOND_TYPE  C3   H5  1
BOND_TYPE  C3   H6  1
BOND_TYPE  C4   H7  1
BOND_TYPE  C4   H8  1
CHI 1  C2   N1   N5   O1
CHI 2  C1   N2   N6   O2
```

```
CHI 3  C4   N3   N7   O3
CHI 4  C3   N4   N8   O4
NBR_ATOM  C2
NBR_RADIUS 5.680783
CHARGE  O1  FORMAL -1
CHARGE  O2  FORMAL -1
CHARGE  O3  FORMAL -1
CHARGE  O4  FORMAL -1
CHARGE  N5  FORMAL 1
CHARGE  N6  FORMAL 1
CHARGE  N7  FORMAL 1
CHARGE  N8  FORMAL 1
ICOOR_INTERNAL    C2    0.000000    0.000000    0.000000   C2    N1    N5
ICOOR_INTERNAL    N1    0.000000  179.999999    1.415354   C2    N1    N5
ICOOR_INTERNAL    N5   -0.000000   61.295484    1.251014   N1    C2    N5
ICOOR_INTERNAL    O1  -90.428667   58.282576    1.169350   N5    N1    C2
ICOOR_INTERNAL    O5  179.954670   59.115765    1.170223   N5    N1    O1
ICOOR_INTERNAL    C1  176.045169   57.691819    1.415219   N1    C2    N5
ICOOR_INTERNAL    N2   86.845895   64.497400    1.415372   C1    N1    C2
ICOOR_INTERNAL    N6  110.556471   61.106762    1.250889   N2    C1    N1
ICOOR_INTERNAL    O2  -96.779676   58.511078    1.169658   N6    N2    C1
ICOOR_INTERNAL    O6 -179.775073   59.004161    1.169290   N6    N2    O2
ICOOR_INTERNAL    C3  173.874084   57.946962    1.415421   N2    C1    N6
ICOOR_INTERNAL    N4   77.799950   64.295527    1.415776   C3    N2    C1
ICOOR_INTERNAL    N8   96.193784   61.170361    1.251280   N4    C3    N2
ICOOR_INTERNAL    O4  -96.296959   58.308011    1.169443   N8    N4    C3
ICOOR_INTERNAL    O8 -179.497702   59.060266    1.169898   N8    N4    O4
ICOOR_INTERNAL    C4  175.291014   57.664682    1.415736   N4    C3    N8
ICOOR_INTERNAL    N3   86.139621   64.127113    1.415269   C4    N4    C3
ICOOR_INTERNAL    N7  110.374813   61.180537    1.251107   N3    C4    N4
ICOOR_INTERNAL    O3  -87.547932   58.501601    1.169613   N7    N3    C4
ICOOR_INTERNAL    O7  179.468303   58.965114    1.169250   N7    N3    O3
ICOOR_INTERNAL    H7 -124.339657   69.732093    1.094071   C4    N4    N3
ICOOR_INTERNAL    H8 -109.797582   70.599597    1.099421   C4    N4    H7
ICOOR_INTERNAL    H5 -125.299685   70.939500    1.093942   C3    N2    N4
ICOOR_INTERNAL    H6 -109.739466   69.419946    1.099264   C3    N2    H5
ICOOR_INTERNAL    H1 -124.303168   69.516839    1.094030   C1    N1    N2
ICOOR_INTERNAL    H2 -109.992752   70.722318    1.099275   C1    N1    H1
ICOOR_INTERNAL    H3  -30.922866   70.848623    1.098998   C2    N1    N5
ICOOR_INTERNAL    H4 -109.962506   69.296989    1.093994   C2    N1    H3
HMXconfsearch.mdb
```

<u>Script used to execute the coupled moves protocol</u>

```
/usr/local/rosetta_bin_linux_2018.26.60275_bundle/main/source/bin/coupled_moves.static.linuxgccrele
ase \
-s structure.pdb \
-nstruct 30 \
-resfile NRdesign.resfile \
-extra_res_fa HMX.params \
-extra_res_fa FMH2.params \
```

```
-coupled_moves::initial_repack false \
-coupled_moves::save_structures true \
-coupled_moves::mc_kt 0.6 \
-coupled_moves::mm_bend_weight 0.1 \
-coupled_moves::ntrials 10000 \
-coupled_moves::ligand_mode true \
-coupled_moves::ligand_prob 0.1 \
-coupled_moves::ligand_weight 2.0 \
-coupled_moves::fix_backbone false \
-coupled_moves::uniform_backrub false \
-coupled_moves::bias_sampling true \
-coupled_moves::bump_check true \
-coupled_moves::trajectory false \
-coupled_moves::trajectory_file traj.pdb \
-coupled_moves::trajectory_stride 500 \
-use_input_sc \
-ex1 \
-ex2 \
-extrachi_cutoff 0
```
